# Long live the king: chromosome-level assembly of the lion (*Panthera leo*) using linked-read, Hi-C, and long-read data

**DOI:** 10.1186/s12915-019-0734-5

**Published:** 2020-01-08

**Authors:** Ellie E. Armstrong, Ryan W. Taylor, Danny E. Miller, Christopher B. Kaelin, Gregory S. Barsh, Elizabeth A. Hadly, Dmitri Petrov

**Affiliations:** 1 Department of Biology, Stanford University, Stanford, CA USA; 2 End2EndGenomics, LLC, Davis, CA USA; 3 Department of Pediatrics, Seattle Children’s Hospital and The University of Washington, Seattle, WA USA; 4 HudsonAlpha Institute for Biotechnology, Huntsville, AL USA; 5 Department of Genetics, Stanford University, Stanford, CA USA

**Keywords:** *Panthera leo*, African lion, Genome assembly, 10x Genomics Chromium, Oxford Nanopore, Dovetail Hi-C, Reference bias, Conservation genomics

## Abstract

**Background:**

The lion (*Panthera leo*) is one of the most popular and iconic feline species on the planet, yet in spite of its popularity, the last century has seen massive declines for lion populations worldwide. Genomic resources for endangered species represent an important way forward for the field of conservation, enabling high-resolution studies of demography, disease, and population dynamics. Here, we present a chromosome-level assembly from a captive African lion from the Exotic Feline Rescue Center (Center Point, IN) as a resource for current and subsequent genetic work of the sole social species of the *Panthera* clade.

**Results:**

Our assembly is composed of 10x Genomics Chromium data, Dovetail Hi-C, and Oxford Nanopore long-read data. Synteny is highly conserved between the lion, other *Panthera* genomes, and the domestic cat. We find variability in the length of runs of homozygosity across lion genomes, indicating contrasting histories of recent and possibly intense inbreeding and bottleneck events. Demographic analyses reveal similar ancient histories across all individuals during the Pleistocene except the Asiatic lion, which shows a more rapid decline in population size. We show a substantial influence on the reference genome choice in the inference of demographic history and heterozygosity.

**Conclusions:**

We demonstrate that the choice of reference genome is important when comparing heterozygosity estimates across species and those inferred from different references should not be compared to each other. In addition, estimates of heterozygosity or the amount or length of runs of homozygosity should not be taken as reflective of a species, as these can differ substantially among individuals. This high-quality genome will greatly aid in the continuing research and conservation efforts for the lion, which is rapidly moving towards becoming a species in danger of extinction.

## Background

The lion (*Panthera leo*) was historically one of the most widespread carnivores on the planet, previously occupying a terrestrial range covering Africa, Europe, and North America [1, 2]. Like most megafauna, the lion is thought to have undergone some declines throughout the Pleistocene, likely due to increased human hunting pressures and climatic changes [1, 2]. However, over just the past 25 years, African lions have lost more than half of their population, while the Asiatic lion has been reduced to fewer than 1000 individuals, occupying little of their former range as a single population in the Gir Forest, India. The remaining Asiatic lions are suspected to be suffering from reproductive declines due to inbreeding depression [3] and have been subject to several outbreaks of canine distemper virus [4].

Genetic markers have played a key role in studying the biogeography, history, and movement of lions for the past 50 years (see, for example [2, 5–10]). However, studies have been mostly limited to microsatellites with limited use of nuclear and mitochondrial sequence data (e.g., [11–17]). More recently, reduced representation sequencing has enabled genomic genotyping using the domestic cat or tiger as a reference [18]. Felid karyotypes are thought to be highly conserved [19, 20], but studies have shown a reference mapping bias for estimation of statistics such as heterozygosity [21] and accurate allele calling [22], both of which are important for assessing population history.

The causes of the decline in lions are multifactorial. Lions have been hunted by humans for thousands of years, possibly first as a direct competitor and threat to survival [23], for initiation rituals and rites of passage [24–26], to reduce predation of domesticated animals, and more recently for sport [27–30]. The illegal trade in lion parts and illicit breeding practices has escalated over the past 10 years, bringing hunting practices and international laws into the spotlight. In addition, several documentaries have exposed the lion breeding industry within South Africa, which uses fenced lions for “petting,” canned hunting experiences, and ultimately as skeletons for export, likely destined for Asian medicines [31]. Accurate and rapid genotyping could aid law enforcement to reveal whether the origins of trafficked goods are from wild or captive populations.

Moreover, rapid population decline has put lions at the forefront of the conservation debate over translocations and how best to manage populations. Many efforts to restore previous populations have focused on translocating lions within and between various South African lion populations (e.g., [32, 33]). Information about local population adaptation, deleterious alleles, and potential inbreeding is lacking, which further complicates managed relocations. While increasing genetic diversity remains a widely accepted conservation goal, recent computer simulations suggest consideration should be made when moving individuals from large heterozygous populations into small homozygous populations [34]. Genomic resources will aid immensely in these estimations and have already shown to be highly preferable to microsatellites or a reduced number of loci (see, for example, [35–37]).

To date, no de novo genome assembly for an African lion exists and only two individuals’ genomes have been resequenced [38]. A de novo assembly of an Asiatic lion was recently completed [39], but as it was limited to short-read technology, is highly fragmented. Asiatic and African lions are currently regarded as separate subspecies [1, 6, 40], and we regard them as such for these analyses. Here, we present a high-quality, de novo genome assembly for the lion (*Panthera leo*), referred to as PanLeo1.0 from a captive female lion, “Brooke,” from the Exotic Feline Rescue Center, Center Point, IN, USA. We use a combination of 10x Genomics linked-read technology, Dovetail Hi-C, and Oxford Nanopore long-read sequencing to build a highly contiguous assembly. We verify the conserved synteny of the lion in comparison with the domestic cat assembly and also examine the demography and heterozygosity of the lion compared with other felids. It is our hope that this genome will enable a new generation of high-quality genomic studies of the lion, in addition to comparative studies across *Felidae*.

## Results

### Genome assembly and continuity

**Fig. 1:**
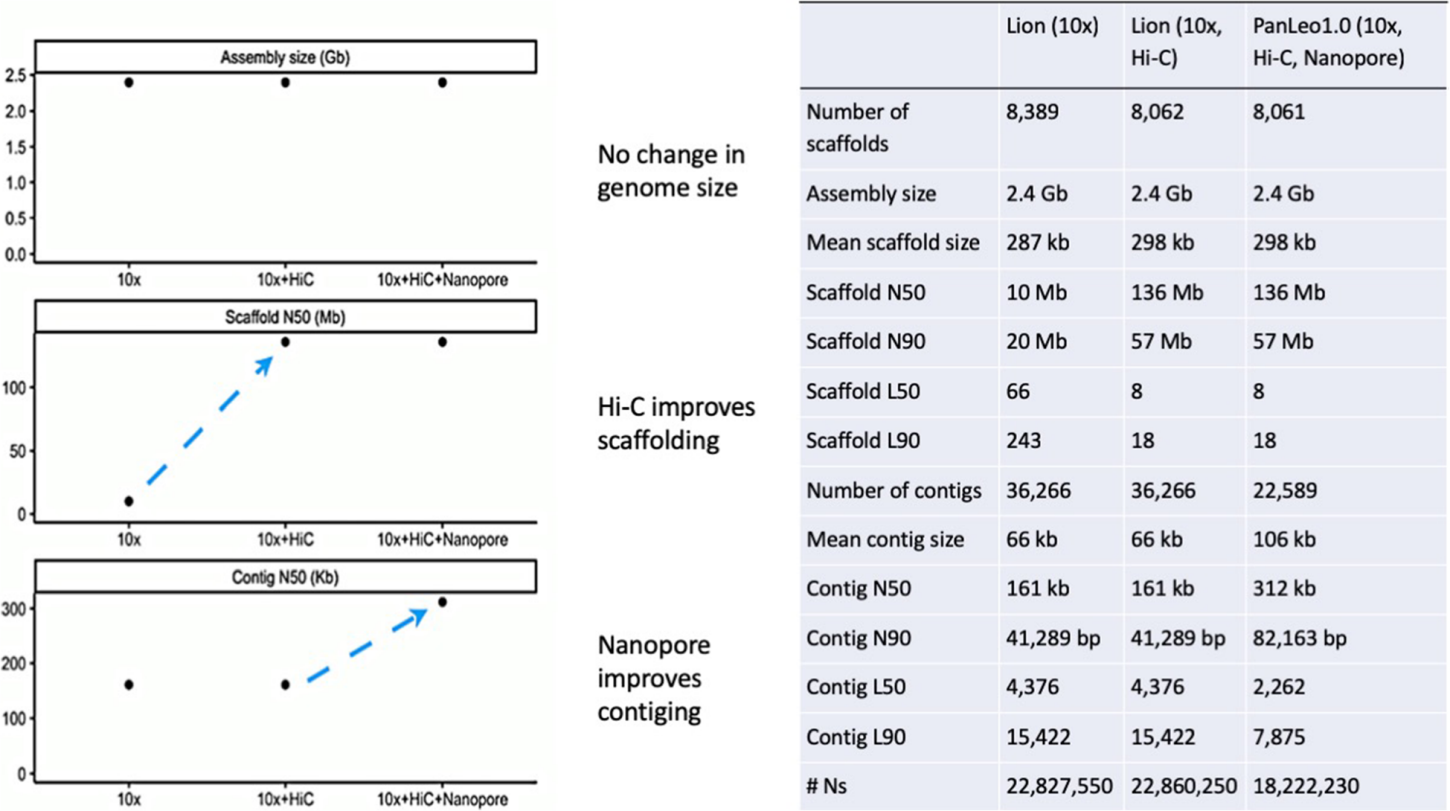
(Left panel) Schematic showing the different improvements made via various technologies in the three assembly phases for the lion genome. (Right panel) Assembly statistics for the three assembly phases of the lion genome

The assembly generated with 10x Genomics Chromium technology yielded a high-quality starting assembly for the lion (Fig. 1). In general, assembly statistics are improved when compared to previous assemblies initially generated using short-insert and mate-pair Illumina libraries, such as the tiger [38], cheetah [41], Amur leopard [42], Iberian lynx [43], and puma [44]. All these assemblies have upgraded their scaffold statistics through a variety of technologies, such as Pacbio, Bionano, Nanopore, or Hi-C (Additional file 1: Table S3; see publications above and DNA Zoo; dnazoo.org). The lower contig scores are consistent with a higher number of missing BUSCO genes (Additional file 1: Tables S4, S5). Although we were unable to compare it to the de novo assembly of the Asiatic lion from Mitra et al. because it has not yet been released publicly, they report a contig N50 of approximately 63 kb, suggesting our assembly represents significant improvement, with a contig N50 of 312 kb (Fig. 1). We then scaffolded the 10x assembly with Dovetail Hi-C, a method which uses chromosomal conformation capture to generate long-rage genomic positioning information (see the “Methods” section for Additional file 2 details). Incorporation of this data resulted in a substantial improvement in the scaffold N50 of the genome (Fig. 1).

### Using long sequencing reads to close gaps in draft genome assemblies

While the draft assemblies using either 10x alone or 10x + Dovetail Hi-C were of high-quality, they contained a number of gaps containing unknown sequence (see #Ns: Fig. 1). We therefore used Oxford Nanopore technology to generate long reads for sequence fill-in. Using a single Oxford Nanopore MinION flowcell, we generated a total of 1,520,012 reads with an average read length of 6528 bp, resulting in approximately 4× coverage of the *P. leo* genome. We then identified single reads which spanned gaps and then, for each gap, used MUSCLE [45] and Cons [46] to generate a consensus sequence spanning that gap (see the “Methods” section). Using this approach, we closed 26,403 gaps of 10, 100, or 400 bp with an average coverage of 3× per gap. Gap sizes were determined automatically, as the 10x Supernova assembler introduces gaps of fixed sizes. We then identified split reads (reads which the aligner split) which spanned any gap 3 kb or larger and again, for any instance in which multiple reads spanned a gap, pooled those reads and used MUSCLE and Cons to generate a consensus sequence spanning the gap. If only one read spanned the gap, the raw sequence from that read was used to fill the gap. This approach resulted in the closing of 574 gaps of 3000, 5000, or 10,000 bp with an average coverage of 1× per gap. Overall, this approach closed 26,977 out of 42,635 gaps on 416 of the 8061 scaffolds in the 10x + Dovetail assembly and reduced the overall size of the genome assembly by 1.6 million bp while increasing the mean contig size from 66 to 106 kb. Overall, this approach resulted in a substantial improvement on average contig size and associated statistics in the lion genome, but did not improve BUSCO scores for the genome. A detailed description of the gaps filled in using Nanopore can be found in Additional file 1: Table S3.

### Phylogenetics

**Fig. 2:**
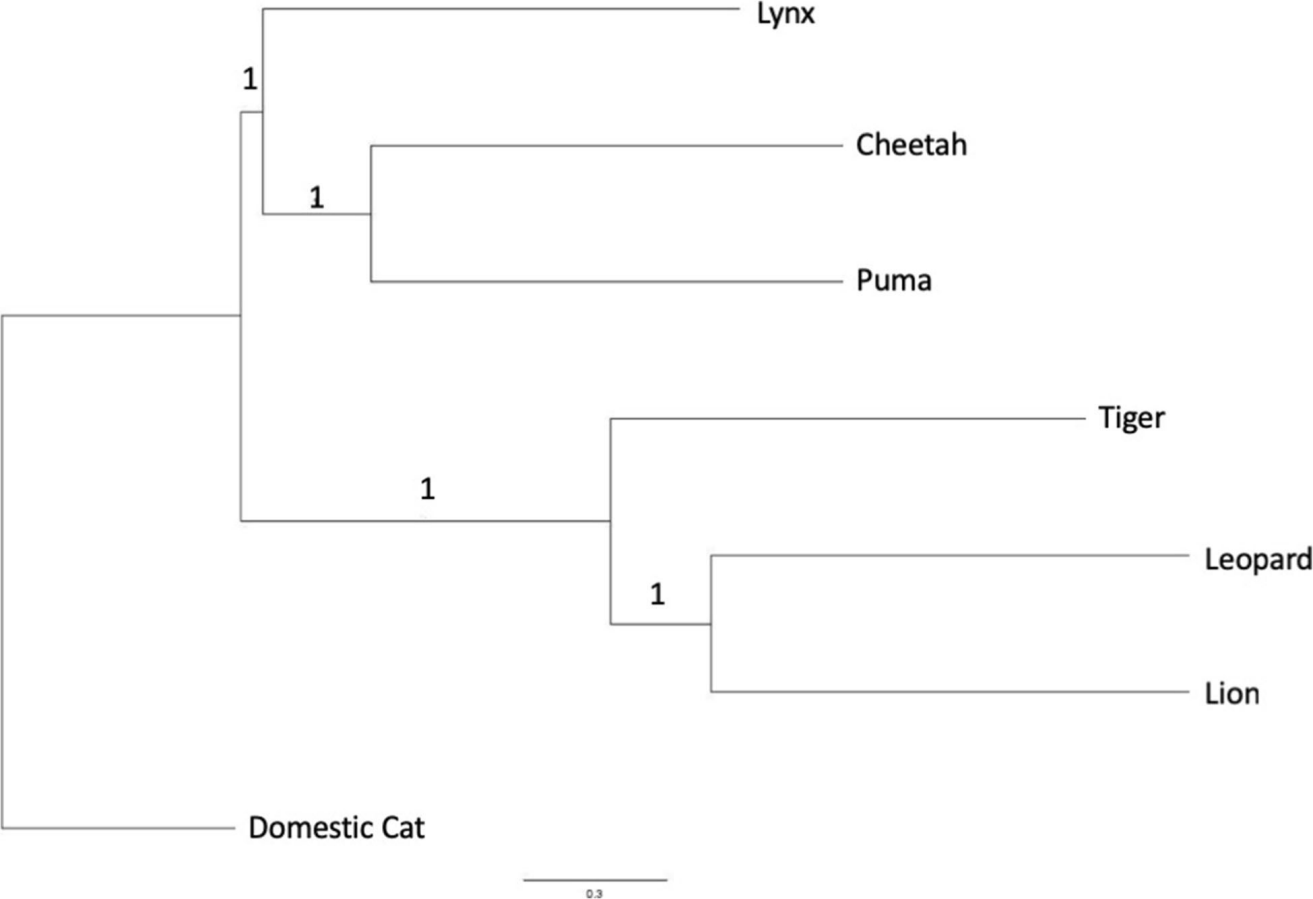
Phylogenetic reconstruction of de novo felid genomes using RAxML and 4039 highly conserved genes from BUSCO mammalia_odb9 dataset. Node annotations indicate posterior probabilities

To verify the phylogenetic relationships of the taxa using the de novo genomes, we constructed a phylogenetic tree using a maximum-likelihood framework using the mammalian gene set from BUSCOv3 to construct a set of individual gene trees with RAxML [47] that were summarized as a species tree using ASTRAL-III (v5.8.3). The domestic cat was manually set as the root for visualization. Consistent with recent phylogenetic analyses of the clade, we found that the lion, the leopard, and the tiger form a cluster representing *Panthera*, with the leopard and lion constituting sister species within the group [48, 49]. The cheetah and puma comprise another cluster, with the lynx sitting outside this grouping [49]. The domestic cat is the most distantly related to all of the species tested here and was used as an outgroup. Since we used protein files (amino acid sequence files derived from BUSCOv3) from the orthologous genes to infer the phylogenetic relationships, we found very high posterior probabilities across all the nodes (Fig. 2).

### Repetitive element and gene annotations

We generated statistics for repetitive elements in each genome using a pipeline which combines homology-based evidence and de novo repeat finding. On average, the continuity of the assembly did not greatly affect our ability to identify repeats (Additional file 1: Table S6). Assemblies from *Panthera* genomes and the domestic cat (Felis_catus_9.0) contained between 40.0 and 42.5% repeats (Additional file 1: Table S7). Alternatively, gene annotation results showed that more continuous assembles generate fewer annotated genes on average (Additional file 1: Tables S8, S9). Possibly, this indicates that more fragmented assemblies cause misidentifications of gene regions by automated annotation software or that genes broken between contigs in more fragmented assemblies are counted multiple times.

### Synteny

**Fig. 3:**
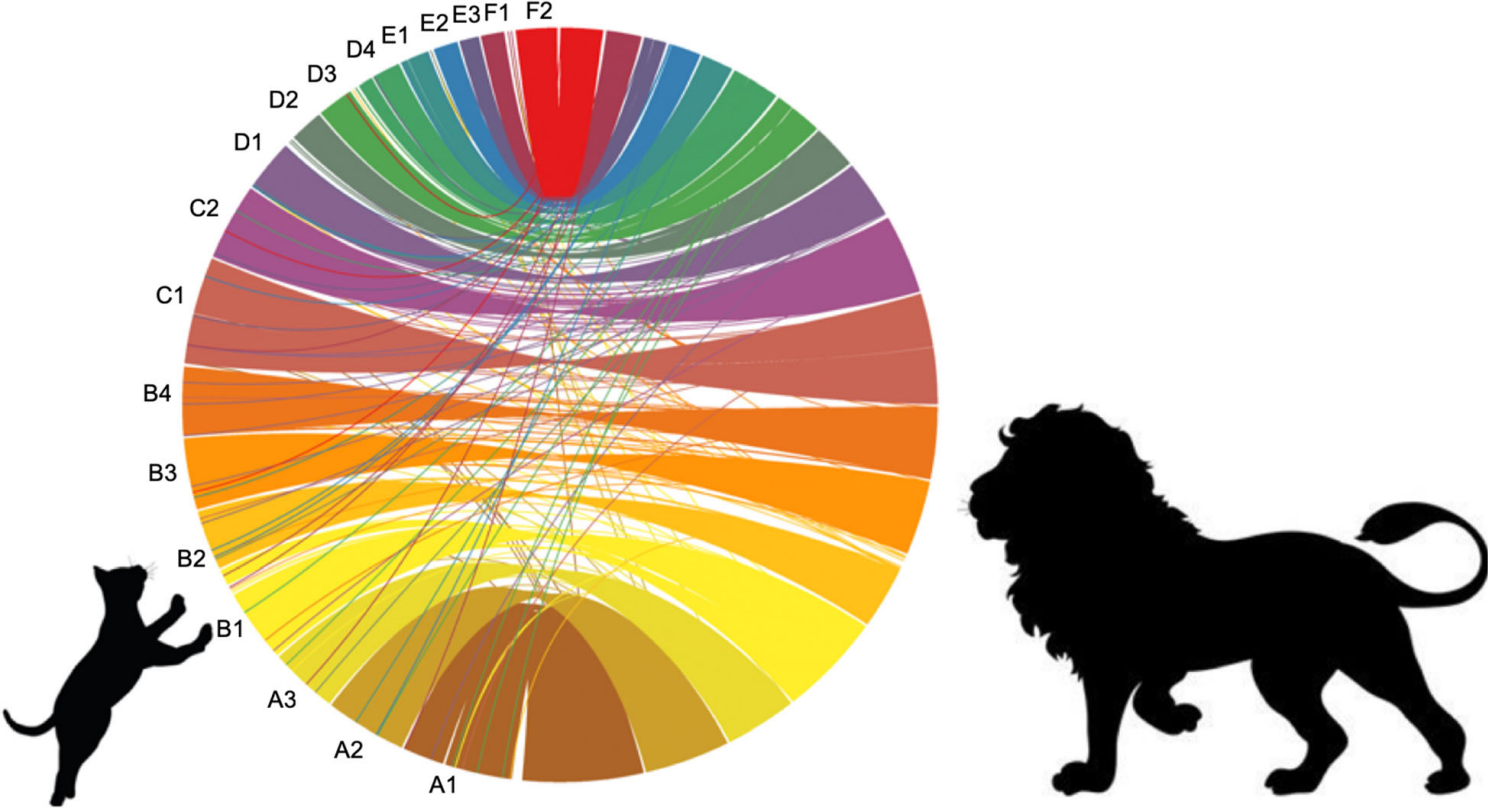
Circos plot of alignments between domestic cat (left) and lion (right) chromosomes. Colors represent different chromosomes indicated by their felCat9 linkage group names (e.g. A1)

We constructed genome synteny visualizations for chromosome-level assemblies of the domestic cat (*F. silvestris*: GCA_000181335), the lion (PanLeo1.0; *P. leo*), and the tiger (*P. tigris* [38, 50, 51];). Each assembly was aligned to the domestic cat and the lion, in order to observe similarities and differences between the genomes. Consistent with expectation due to the stable karyotype (chromosome number and visual appearance) of extant *Felidae* [19, 20, 52], we found very few rearrangements in the karyotype across species (Fig. 3, Additional file 1: Figures S1, S2).

### Heterozygosity

**Fig. 4:**
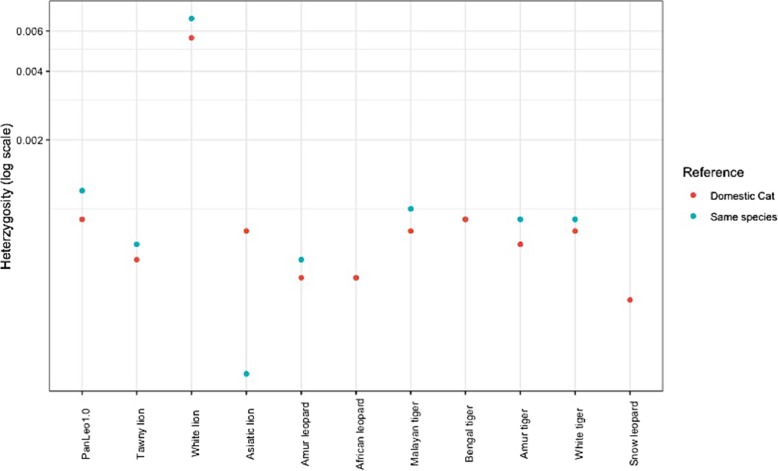
Average genome-wide heterozygosity of various felids when mapped to a reference genome from their own species, if available (blue) vs. when mapped to the domestic cat (red)

We mapped raw Illumina reads to each respective species genome, as well as to the domestic cat assembly. We found that on average, mapping to the domestic cat assembly resulted in lower heterozygosity calls and an average of 10% fewer reads successfully mapped (Additional file 1: Table S11). However, this pattern was inconsistent and reversed for the Asiatic lion individual (Fig. 4, Additional file 1: Table S11). These results are supported by Gopalakrishnan et al. [21], who found that the reference used had some effect on heterozygosity inference, but little effect on the inference of population structure. In addition, we find that there is substantial variation in genome-wide heterozygosity estimates across the four lions that were tested (PanLeo1.0, 0.0012; Tawny lion, 0.0007; White lion, 0.007; and Asiatic lion, 0.00019). The two captive lions sequenced in Cho et al. may have been substantially inbred or outbred in captivity, but no further details on the individuals are available.

Because the assembly quality varied, we also tested whether reference genome continuity had an effect on heterozygosity calls (Additional file 1: Table S10). We find that in general, more fragmented assemblies do not seem to strongly influence heterozygosity calls (Additional file 1: Table S10).

### Runs of homozygosity

Using the mapped files created during the previous step, we investigated how runs of homozygosity (ROH) were distributed across the four lion genomes. We found that there were a high proportion of relatively short runs (10–100kb) of homozygosity contained within the Asiatic lion genome (Additional file 1: Figures S3, S4, Table S12), and to a lesser extent, the two previously published captive lion genome sequences from Cho et al. In general, heterozygosity was much lower genome-wide in the Asiatic individual (Additional file 1: Figures S3, S4), indicating that along with showing signs of recent inbreeding, the population has likely been small for a long time (see [53])*.*

**Fig. 5:**
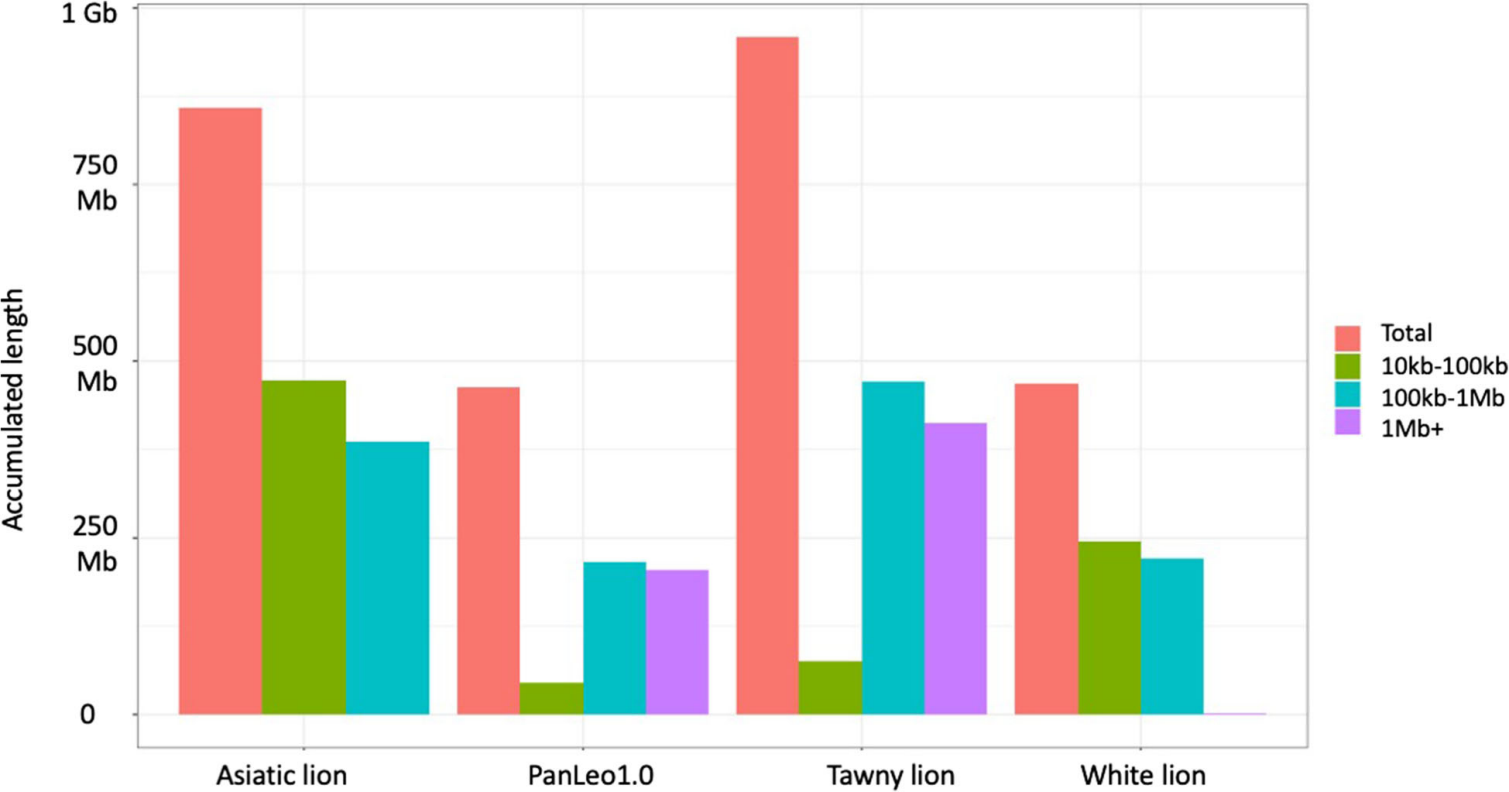
Distribution of lengths of homozygosity across various lion individuals

When the lengths of runs of homozygosity were divided into different length categories (10–100 kb, 100 kb–1 Mb, and 1 Mb or greater), it was observed that the tawny lion from Cho et al. had the greatest amount of the total genome in ROH, followed by the Asiatic lion, then the white lion, and last the lion from this study (Fig. 5), “Brooke.” Interestingly, the tawny lion also had most of its genome in ROH of length 1 Mb or greater, followed by “Brooke,” which indicated very recent inbreeding, but both of these genomes had very few short runs of ROH in the 10–100kb window (Fig. 5, Additional file 1: Table S12). The Asiatic lion, which is from a population known for the potential to be inbred due to rapid declines, did not have any portion of its genome in a run greater than 1 Mb (Fig. 5, Additional file 1: Table S12). This could be due to recent efforts by managers to protect and expand the remaining Asiatic lions (reviewed in [54]), and the large portion of the genome in intermediate ROH runs (10–100kb and 100 kb–1 Mb) may be reflective of the previous and rapid population decline.

### Demographic history

**Fig. 6:**
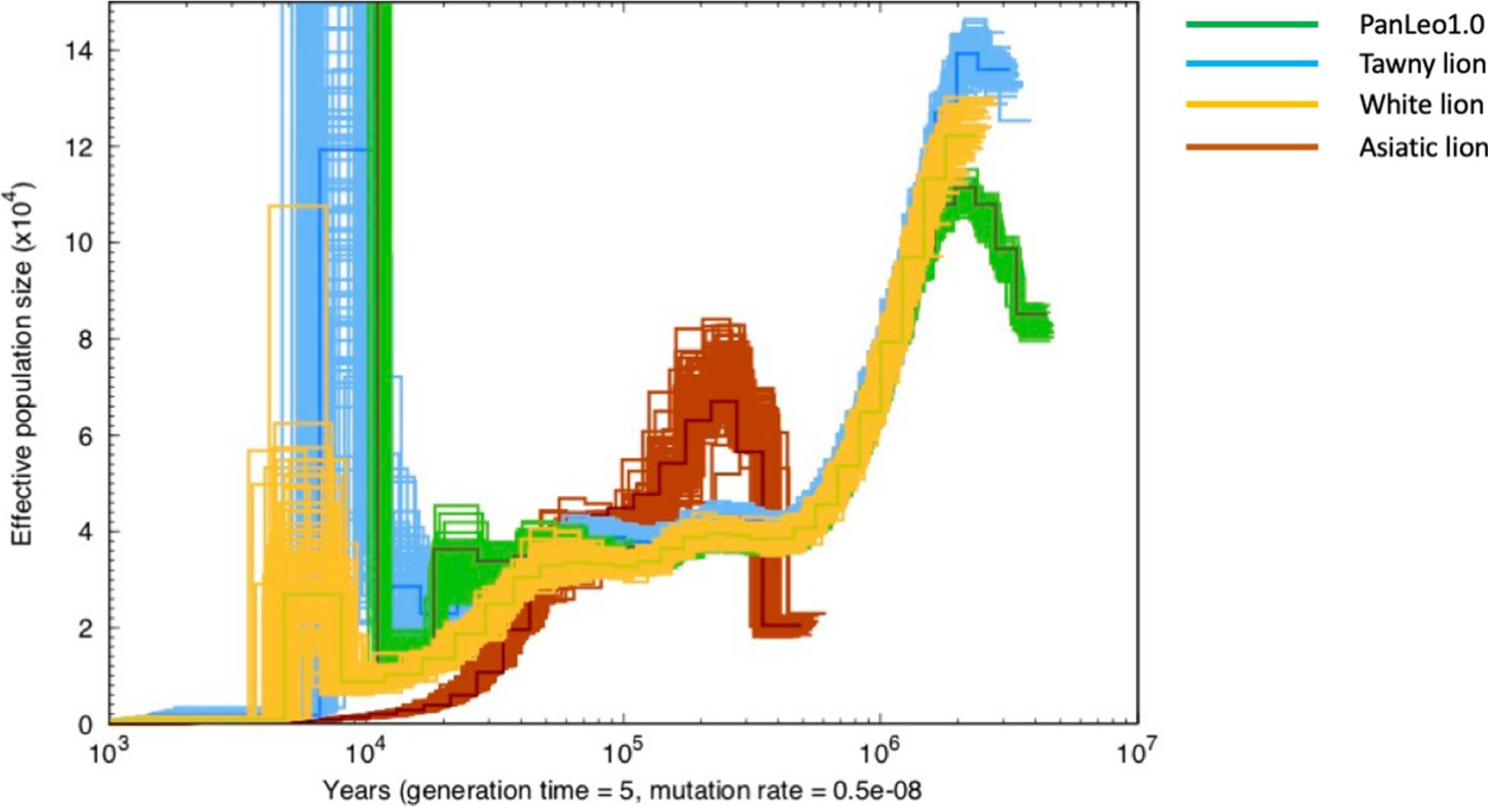
Demographic history of the lion as inferred by PSMC, with the PanLeo1.0 used as the reference genome. Generation time used was 5 years, and mutation rate applied was 0.5 × 10^−8^

**Fig. 7:**
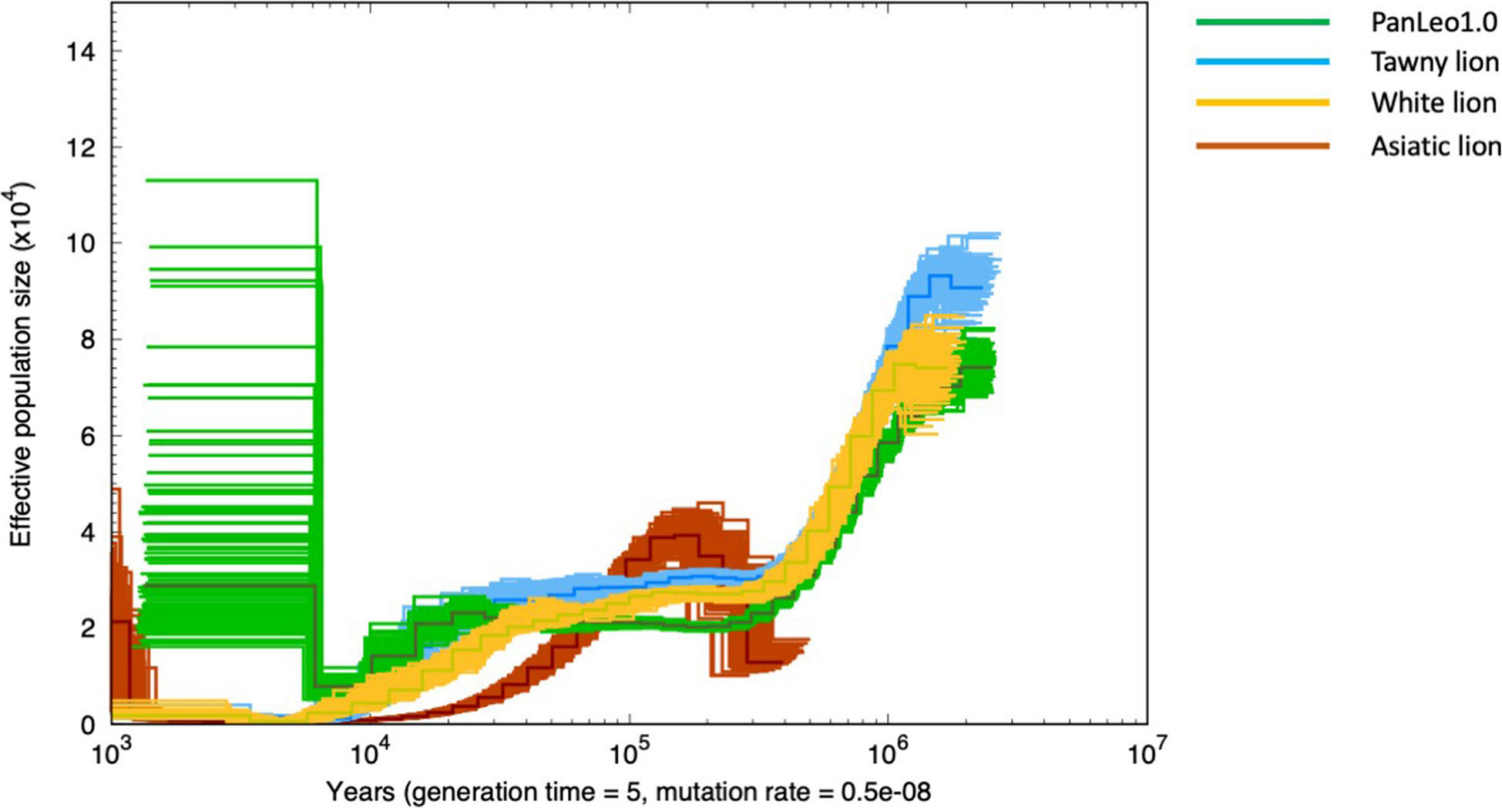
Demographic history of the lion as inferred by PSMC, with felCat9 used as the reference genome. Generation time used was 5 years, and mutation rate applied was 0.5 × 10^−8^

PSMC (Pairwise Sequential Markovian Coalescent) analyses revealed similar demographic histories of PanLeo1.0 and the two genomes from Cho et al. (“Tawny lion” and “White lion”; Figs. 5 and 6). These genomes show an initial decline approximately 2 million years ago (MYA) and a second decline beginning nearly 50,000 years ago (Figs. 6 and 7). Declines in the three putative African lions (PanLeo 1.0, Tawny lion, and White lion) starting 2MYA likely represent the emergence of the modern lion species (from a larger meta-population of ancient lions), which is supported by both fossil evidence [55] and dating estimates of the *Panthera* clade [48, 49]. These trends are consistent with the fossil record which has revealed declines of large mammal populations during this time period, possibly due to Archaic human influence and/or climate changes (e.g., [56, 57]). The Asiatic lion genome shows a more rapid decline over the past 100,000 years and a substantially shorter period of stabilization around 100,000 compared to the African lion. It is possible that the low heterozygosity of the Asiatic lion was low enough to impede the inference of accurate historical N_E_ due to a distortion of the coalescent patterns across the genome. Corroborating these issues, other studies have shown variation between results in PSMC analyses within individuals of the same species and suggest that alternative coalescent methods should be used to confirm historical demographic trends [58]. PSMC analyses also showed differences in the predictive effective population size when using either PanLeo1.0 as a reference (Fig. 6) or felCat9 (version 9.0 domestic cat reference assembly) as a reference (Fig. 7). We found no substantial difference in the trajectory of effective population size of PanLeo1.0 when using a generation time of 6 years (Additional file 1: Figure S5).

The spike in N_E_ observed for both PanLeo1.0 and the Tawny lion (approximately 104 years ago; Fig. 6) also suggests that these individuals are the result of relatively recent admixture between populations. However, interestingly, this signal disappears when mapping both individuals to the domestic cat. The loss of signal may be a reflection on the software’s ability to detect heterozygous sites, and thus the inference of N_E_ over time, when mapping to different reference sequences. We were able to show this bias when investigating heterozygosity signals across different references using ANGSD, but BCFtools (which was used to identify heterozygous sites prior to PSMC) may have different sources of bias. Given the development of new methods to extend and improve PSMC, it would be pertinent to investigate the sources of bias (e.g., sequence quality, mapping score, depth) across difference software and its outcome on theta and N_E_ estimation.

## Conclusions

Linked-read, long-read, and long-range scaffolding genomic technologies such as 10x Genomics, Nanopore, and Hi-C allow rapid and economical de novo construction of high-quality and highly contiguous genomes (e.g., [59]). Projects such as Genome 10k [60, 61], i5k [62], DNA Zoo (dnazoo.org [50, 51];), and Bird 10k [63] aim to vastly improve our general understanding of the evolution of genomes, and both the origin and fate of diversity of life on Earth. Such high-quality assemblies will not only contribute to our understanding of the evolution of genomes, but also have practical applications in population genetics and conservation biology.

The chromosome-level de novo assembly of the lion genome presented here was constructed in three steps—10x Genomics was used to create the base assembly, and Dovetail Hi-C and Oxford Nanopore were used to improve contiguity. We show that each step results in substantial improvement to the genome, indicating that these methods are not redundant. At the same time, our data indicate that 10x and Hi-C alone are enough to approximate chromosomes in a typical mammal genome. Nanopore data, even with a small amount of very long reads, was enough to fill in many of the small gaps and ambiguous sequences across the genome.

The quality of this assembly allowed us to investigate the co-linearity of the genome compared to other felids and the importance of the reference sequence for estimating heterozygosity. As has been reported before [19, 20], we find that the genomes of felids are largely co-linear and indicate that no large-scale chromosomal rearrangements have occurred across species. However, reference sequence bias can have substantial and unpredictable effect on estimating heterozygosity, possibly due to mismapping. Our results suggest that there may be a substantial variation of heterozgyosity inference between methods, especially those which require this calculation as part of the underlying inference, such as with BCFtools ROH and PSMC. More investigation into the underlying causes and consequences of these differences would be pertinent.

The variation of heterozygosity inference across the four lions tested here is further evidence that single genomes are not representative of the heterozygosity of a species or even the populations (captive or wild) from where they are derived. This assembly has also allowed us to compare fine-scale patterns of heterozygosity and runs of homozygosity across the genome, where we find a substantial amount of variation between individuals. This contiguous genome will allow us to perform analyses on recent inbreeding and ROH in wild individuals across their range, how heterozygosity patterns differ between populations with different evolutionary histories, and how management decisions such as translocations and barriers to dispersal affect wild populations. Further, captive management of populations also stand to gain from genetic monitoring tools, and as we have shown here, individuals from zoos may harbor early signs of diversity loss and the accumulation of long runs of homozygosity. Even outside the nuanced case of the Asiatic lion, where dramatic population declines occurred prior to managers stepping in to monitor individuals, captive-bred populations often come from few founders with the addition of new individuals as available. If captive populations are truly meant to be a resource for conservation at large, more work must be done to understand the genetic implications of such scenarios.

Demographic analyses are also greatly aided by continuous sequence and rely on the inference of coalescence across the genome. As we detected a different historic demography for the Asiatic lion, it would be pertinent to examine how recent and rapid inbreeding affects the ability of these software to detect N_E_ over time. Further, examination of the patterns of diversity loss across wild individuals, especially populations which have been suggested to show signs of inbreeding (see the Ngorongoro crater lion population [3, 10, 64];), will aid managers in decision-making to ensure a future for existing lion populations. Further, additional investigations should be made into how the use and choice of reference genome impacts demographic history prediction and whether these different estimates are a reflection of reference bias.

This study should aid in conservation efforts for the lion and enable studies across many facets of evolutionary biology, such as improving our knowledge of possible hybridization across the genus *Panthera*, or the basis of their phenotypic diversity. Undeniably, lion research has a historic legacy of collaboration across fields [65] and this genome will aid in future endeavors to prevent further loss of one of the world’s most iconic species. Most importantly, it will enable low-cost resequencing efforts to be completed, in addition to a wide range of other genetic studies, in order to further the conservation efforts of the lion.

## Methods

### Library preparation and sequencing

Whole blood samples were collected on two occasions during routine dental and medical procedures on an adult female lion (“Brooke”) from the Exotic Feline Rescue Center (Center Point, IN, USA) in 2017. Blood was collected in EDTA tubes, briefly held at − 20 °C before being shipped overnight to Stanford University and subsequently frozen at − 80 °C. Approximately 200 μL of whole blood was used for 10x Genomics Chromium library preparation and sequencing at HudsonAlpha in Huntsville, AL. Briefly, DNA was extracted from the whole blood sample using the Qiagen MagAttract HMW DNA Kit. Procedures were altered slightly according to the recommendations made by 10x Genomics, which are detailed on their site (https://support.10xgenomics.com/de-novo-assembly/sample-prep/doc/demonstrated-protocol-hmw-dna-extraction-from-whole-blood). This library was sequenced on an Illumina HiSeq X Ten. An additional 1 mL of EDTA collected whole blood was then sent to Dovetail Genomics in Santa Cruz, CA, for Hi-C library preparation and subsequent sequencing on the Illumina HiSeq X Ten platform. Briefly, two libraries were prepared in a similar manner as previously described (Lieberman-Aiden et al.). Briefly, chromatin was fixed in place with formaldehyde in the nucleus and then extracted. Fixed chromatin was digested with DpnII, the 5′ overhangs filled in with biotinylated nucleotides, and then free blunt ends were ligated. After ligation, crosslinks were reversed and the DNA purified from protein. Purified DNA was treated to remove biotin that was not internal to ligated fragments. The DNA was then sheared to ~ 350 bp mean fragment size, and sequencing libraries were generated using NEBNext Ultra enzymes and Illumina-compatible adapters. Biotin-containing fragments were isolated using streptavidin beads before PCR enrichment of each library. The libraries were sequenced on an Illumina HiSeq X Ten platform. The number and length of read pairs produced for each library was 208 million, 2 × 150 bp for library 1, and 220 million, 2 × 150 bp for library 2. Together, these Dovetail Hi-C library reads provided approximately 24× physical coverage of the genome.

DNA for Nanopore sequencing was extracted from three 500 μL aliquots of whole blood using the Quiagen DNeasy kit following the manufacturer’s instructions. DNA was eluted into 50 μL and then concentrated to approximately 25 ng/μL using a Zymo DNA Clean and Concentrator Kit. The final elution volume after concentrating was approximately 50 μL. Libraries for Nanopore sequencing were prepared using a 1D genomic ligation kit (SQK-LSK108) following the manufacturer’s instructions with the following modifications: dA-tailing and FFPE repair steps were combined by using 46.5 μL of input DNA, 0.5 μL NAD+, 3.5 μL Ultra II EndPrep buffer and FFPE DNA repair buffer, and 3.0 μL of Ultra II EndPrep Enzyme and FFPE Repair Mix, for a total reaction volume of 60 μL. Subsequent thermocycler conditions were altered to 60 min at 20 °C and 30 min at 65 °C. The remainder of the protocol was performed according to the manufacturer’s instructions. Fifteen microliters of the resulting library was loaded onto a MinION with a R9.4.1 flowcell and run for 48 h using MinKNOW version 2.0. Fastq files were generated from raw Nanopore data using Albacore version 2.3.1. Pass and fail reads were combined for a total of 1,520,012 reads with an average read length of 6528 bp, with 336,792 of these reads greater than 10 kb, and a longest read length of 62,463 bp.

### Genome assembly

The 10x reads were assembled using Supernova version 1.2.1 with standard settings [66]. A single haplotype of the genome was output using the “--pseudohap 1” flag. This assembly was then provided to the HiRise software [67] as the starting assembly. The input de novo assembly, shotgun reads, and Dovetail Hi-C library reads were used as input data for HiRise, a software pipeline designed specifically for using proximity ligation data to scaffold genome assemblies (Putnam et al. 2016). Shotgun and Dovetail Hi-C library sequences were aligned to the draft input assembly using a modified SNAP read mapper (http://snap.cs.berkeley.edu). The separations of Dovetail Hi-C read pairs mapped within draft scaffolds were analyzed by HiRise to produce a likelihood model for genomic distance between read pairs, and the model was then used to identify and break putative misjoins, to score prospective joins, and make joins above a threshold. After scaffolding, shotgun sequences were used to close gaps between contigs. All Hi-C assembly steps were performed by Dovetail Genomics (Santa Cruz, CA), and the resulting assembly returned to us.

### Using long sequencing reads to close assembly gaps

Long sequencing reads generated by Nanopore sequencing were used to close gaps in the 10x + Dovetail assembly. First, all Nanopore reads were mapped to the 10x + Dovetail Hi-C assembly using BWA [68] with the ont2d option (flags: -k14 -W20 -r10 -A1 -B1 -O1 -E1 -L0). Gaps were then closed using one of two methods. We first identified single reads that had not been split by the aligner that mapped to at least 50 bp of sequence on either side of a gap in the 10x + Dovetail assembly and found 110,939 reads meeting this criteria. The sequence spanning the gap plus 50 bp on either side was extracted from the read and combined with other reads spanning the same gap into a single fasta file. To improve the quality of the alignment, 50 bp of sequence from either side of the gap from the reference genome was added to the fasta file. MUSCLE version 3.8.31 [46] was used, with default settings, to generate a multiple sequence alignment using all input sequences for each gap. Cons version 6.5.7.0 [45] was used to create a consensus sequence from the multiple alignment generated by MUSCLE. Nucleotide positions at which Cons could not determine a highest scoring residue were removed.

Gaps not closed by single reads were then filtered, and instances in which a single read was split and mapped to either side of a gap were identified, revealing 841 reads meeting these criteria. The sequence that spanned the gap but was not mapped was isolated, and the 50 bp of sequence from the reference genome was added to either side of the unmapped sequence in a fasta file containing all gaps. In those instances where more than one split read spanned a gap, MUSCLE was used to generate a multiple sequence alignment and Cons was then used to create a consensus sequence. Gaps in the reference genome were then replaced with the new consensus sequence.

### Assessment of assembly quality

In order to assess the continuity of each genome assembly, we first ran scripts from Assemblathon 2 (assemblathon_stats.pl; https://github.com/ucdavis-bioinformatics/assemblathon2-analysis), which gives a detailed view of the contig and scaffold statistics of each genome [69]. We then ran BUSCOv3 [70] in order to assess the conserved gene completeness across the genomes. We queried the genomes with the mammalian_odb9 dataset (4104 genes in total). We ran all three versions of the genome assembled here (10x, 10x + Hi-C, and 10x + Hi-C + Nanopore). The final version of the assembly (10x + Hi-C + Nanopore) is what we refer to as PanLeo1.0.

### Phylogeny estimation

We also used the genes queried by BUSCOv3 in order to infer phylogenetic relationships among *Panthera* (see Additional file 1: Table S1 for details of sequences and genomes used). We first extracted all the genes in the mammalia_odb9 dataset produced for each genome, in addition to the domestic cat genome assembly (felCat9) by each independent BUSCO run, which totaled 4039 genes. These protein sequences were then aligned using MAAFT ([71]; flags “--genafpair” and “--maxiterate 10000”). We then used RAxML [47] to build phylogenies for each of the genes. We used flags “-f a,” “-m PROTGAMMAAUTO,” “-p 12345,” “-x 12345,” and “-# 100,” which applied a rapid bootstrap analysis (100 bootstraps) with a GAMMA model for rate heterogeneity. Flags “-p” and “-x” set the random seeds. We subsequently used the “bestTree” for each gene and ran ASTRAL-III (v5.6.3) on the 100 bootstrap replicates for each gene produced by RAxML [72] on the resulting trees (3439 trees total) to output the best tree under a maximum-likelihood framework. By default, ASTRAL-III performs 100 bootstrap replicates on the input.

### Repeat masking

We identified repetitive regions in the genomes in order to perform repeat analysis and to prepare the genomes for annotation. Repeat annotation was accomplished using homology-based and ab initio prediction approaches. We used the felid RepBase (http://www.girinst.org/repbase/ [73];) repeat database for the homology-based annotation within RepeatMasker (http://www.repeatmasker.org [74];). The RepeatMasker setting -gccalc was used to infer GC content for each contig separately to improve the repeat annotation. We then performed ab initio repeat finding using RepeatModeler (http://repeatmasker.org/RepeatModeler.html [75];). RepeatModeler does not require previously assembled repeat databases and identifies repeats in the genome using statistical models. We performed two rounds of repeat masking for each genome. We first hard masked using the “-a” option and “-gccalc” in order to calculate repeat statistics for each genome. We subsequently used the “-nolow” option for soft-masking, which converts regions of the genome to lower case letters (a, c, g, t), but does not entirely remove them. The soft-masked genome was used in subsequent genome annotation steps.

### Annotation

Gene annotation was performed with the Maker3 annotation pipeline using protein homology evidence from the felid, human, and mouse UniProt databases. Gene prediction was performed with Augustus [76] and trained using human gene models. We calculated annotation statistics on the final “gff” file using jcvi tools “-stats” option [77].

### Synteny

We identified scaffolds potentially corresponding to chromosomes and any syntenic re-arrangements between species. To do this, we used the LAST aligner [78] to align the 20 largest scaffolds from each assembly to the linkage groups established by felCat9 (NCBI: GCA_000181335). We first created an index of each genome using the “lastdb” function with flags “-P0,” “-uNEAR,” and “-R01.” We then determined substitutions and gap frequencies using the “last-train” algorithm with flags “-P0,” “--revsym,” “--matsym,” “--gapsym,” “-E0.05,” and “-C2.” We then produced many-to-one alignments using “lastal” with flags “-m50,” “-E0.05,” and “-C2,” and the algorithm “last-split” with flag “-m1.” Many-to-one alignments were filtered down to one-to-one alignments with “maf-swap” and “last-split” with flag “-m1.” Simple sequence alignments were discarded using “last-postmask,” and the output converted to tabular format using “maf-convert -n tab.” Alignments were then visualized using the CIRCA software (http://omgenomics.com/circa), and mismap statistics calculated. We did not visualize any alignments that had an error probability greater than 1 × 10^−5^. We additionally did not plot the sex chromosomes due to excessive repetitive regions and differences between the sexes of the animals that we used.

### Heterozygosity

Raw Illumina reads from each species were mapped to the domestic cat genome (NCBI: GCA_000181335) and the reference genome for each respective species using BWA-MEM [68]. Observed heterozygosity was calculated using ANGSDv0.922 [79]. We first estimated the site frequency spectrum (SFS) for single samples using the options “-dosaf 1,” “-gl 1,” “-anc,” “-ref,” “-C 50,” “-minQ 20,” “-fold 1,” and “-minmapq 30” (where “-anc” and “-ref” were used to specify the genome it was mapped to). Subsequently, we ran “realSFS” and then calculated the heterozygosity as the second value in the site frequency spectrum.

To control for possible differences in heterozygosity due to mapping or assembly quality, we also performed the same analysis on genome assemblies of different qualities for the lion (*P. leo*; this study, 10x and 10x + Hi-C + Nanopore), and the tiger (*P. tigris* [38, 50, 51, 80];).

### Runs of homozygosity

Mapped sequences subsequently were used to infer runs of homozygosity across the genome. We used the “mafs” output files from an additional run using ANGSD by adding the filters “-GL 1,” “-doMaf 2,” “-SNP_pval 1e-6,” “-doMajorMinor 1,” “-only_proper_pairs 0,” and “-minQ 15.” This run outputs a file that contains the positions of heterozygous sites across the genome. We counted the number of heterozygous sites in 1 Mb bins across each scaffold and computed (1) the number of heterozygous sites in each bin and (2) the frequency of bins containing the number of heterozygous sites per kilobase. We then visualized this across the chromosomes as a proxy for runs of homozygosity in the genome. One megabase bin sizes were chosen as an arbitrary, but likely intermediate length run of homozygosity.

Further, we used BCFtoolsv1.9 (Narasimhan et al.) to estimate the length of runs of homozygosity. We restricted this analyses to autosomal scaffolds identified during the mapping stage by using SAMtools view on each mapped file. Traditional variant call files (VCF) were generated using bcftools mpileup with flags “-Ou” and subsequently BCFtools call with flags “--skip-variants indels,” “-Ov,” and “-mv.” Indels were skipped during this step because genotype calls in these regions tend to be enriched for errors due to low mapping quality and mismaps. We filtered these files for sites with greater than a depth of 10× depth and with a quality score over 20, using BCFtools “filter” with flags “-i DP > 10&QUAL> 20.” Subsequently, we ran BCFtools RoH with flags “-G 30” and “--AF-dflt 0.4” to specify the use of genotype calls with a quality of 30 or more and to set a default allele frequency, since the allele frequencies of these populations are unknown.

### Demographic history

We mapped all data to the genome assemblies of both PanLeo1.0 and felCat9. Subsequently, only autosomal scaffolds were retained using the SAMtools (for PanLeo1.0, only the major scaffolds identified as autosomes in the previous section on synteny were retained). The remaining scaffolds were used for Pairwise Sequential Markovian Coalescent (PSMC) [81]. Reads were mapped to the remaining scaffolds using BWA-MEM [68], and the consensus sequence called using SAMtools mpileup [82], BCFtools call, and vcfutils “vcf2fastq.” Minimum depth cutoffs of 10 and maximum depth cutoffs of 100 were applied to all genomes using vcfutils. In order to visualize the PSMC graphs, we applied a mutation rate of 0.5e−08 [38] and a generation time of 5 years for the lion [38]. We compared these inferences with those from two previously resequenced lions [38] and the Asiatic lion [39]. We additionally tested a generation time of 6 years because there have been contrasting estimates of generation time for lions (see https://www.iucnredlist.org/species/15951/115130419). However, we use 5 years in the main text in order to be consistent with previous demographic estimates.

## Supplementary information

**Additional file 1: Table S1.** Summary of data sources used for analysis. **Table S2.** Details of genome assembly fill in with Oxford Nanopore data. **Table S3.** Comparative assembly statistics from Assemblathon 2 scripts [69] from published *Panthera* and *Felid* genomes. **Table S4.** BUSCOv3 scores for assembly completeness of the three assembly phases of the African lion genome. **Table S5.** Comparative BUSCO scores between *published* Panthera *and Felid* assemblies. **Table S6.** Repeat element statistics for the three lion de novo genome assemblies generated in this study. **Table S7.** Repeat element statistics for various Panthera assemblies and the domestic cat. **Table S8.** Annotation statistics for the three lion de novo assemblies generated in this study from the JCVI program. **Table S9.** Annotation statistics for *Panthera* genome assemblies and the domestic cat (felCat9) using jcvi. **Table S10.** Observed heterozygosity statistics from various assembly versions of the lion (mapped to the “10x only” and “PanLeo1.0) and tiger (from Cho et al. 2013, and the upgraded DNA Zoo tiger assembly). **Table S11.** Heterozygosity (observed) from various *Panthera* individuals when mapped to respective species genome (i.e. lions were mapped to PanLeo1.0, tigers mapped to DNAZoo tiger assembly) genome compared to when mapped to the domestic cat. **Table S12.** Lengths of runs of homozygosity across various lion genomes using PanLeo1.0 as reference. **Figure S1. Figure S2.** Circos plot of alignments between tiger (right) and domestic cat (left) chromosomes. Colors represent different chromosomes with bottom chromosome (shown in dark brown) representing A1. **Figure S3.** Histograms of per window heterozygosity. Graphs skewed more left represent individuals with more windows having lower heterozygosity on average. A: Lion from this study, PanLeo1.0, B: Tawny lion, Cho et al. (2013), C: White lion, Cho et al. (2013), D: Asiatic lion, Mitra et al. (2019). **Figure S4.** Genome-wide heterozygosity. Panels show heterozygosity genome-wide in non-overlapping 1 Mb bins. A: Lion from this study, PanLeo1.0, B: Tawny lion, Cho et al. (2013), C: White lion, Cho et al. (2013), D: Asiatic lion, Mitra et al. (2019). Red line represents the mean heterozygosity value genome-wide. **Figure S5.** Bootstrap PSMC plot comparing generation times of 5 and 6 years using PanLeo1.0 as the reference sequence.

**Additional file 2.** Various code used to map and analyze results described in manuscript.

## Data Availability

Raw reads for genome sequencing have been deposited at NCBI Short Read Archive (SRA) under BioProject number PRJNA556895 [83]. Genome assembly has been deposited at NCBI under BioProject number PRJNA556895 [83]. All other intermediate data files and scripts are available from the corresponding authors upon request.
