# Supplementary material for: Long live the king: chromosome-level assembly of the lion (Panthera leo) using linked-read, Hi-C, and long-read data
Source: BMC Biol. 2020 Jan 8;18:3. doi: 10.1186/s12915-019-0734-5 (PMC6950864; doi:10.1186/s12915-019-0734-5)
Supplement: Supplementary file 1 — Additional file 1: Table S1. Summary of data sources used for analysis. Table S2. Details of genome assembly fill in with Oxford Nanopore data. Table S3. Comparative assembly statistics from Assemblathon 2 scripts [69] from published Panthera and Felid genomes. Table S4. BUSCOv3 scores for assembly completeness of the three assembly phases of the African lion genome. Table S5. Comparative BUSCO scores between published Panthera and Felid assemblies. Table S6. Repeat element statistics for the three lion de novo genome assemblies generated in this study. Table S7. Repeat element statistics for various Panthera assemblies and the domestic cat. Table S8. Annotation statistics for the three lion de novo assemblies generated in this study from the JCVI program. Table S9. Annotation statistics for Panthera genome assemblies and the domestic cat (felCat9) using jcvi. Table S10. Observed heterozygosity statistics from various assembly versions of the lion (mapped to the “10x only” and “PanLeo1.0) and tiger (from Cho et al. 2013, and the upgraded DNA Zoo tiger assembly). Table S11. Heterozygosity (observed) from various Panthera individuals when mapped to respective species genome (i.e. lions were mapped to PanLeo1.0, tigers mapped to DNAZoo tiger assembly) genome compared to when mapped to the domestic cat. Table S12. Lengths of runs of homozygosity across various lion genomes using PanLeo1.0 as reference. Figure S1. Figure S2. Circos plot of alignments between tiger (right) and domestic cat (left) chromosomes. Colors represent different chromosomes with bottom chromosome (shown in dark brown) representing A1. Figure S3. Histograms of per window heterozygosity. Graphs skewed more left represent individuals with more windows having lower heterozygosity on average. A: Lion from this study, PanLeo1.0, B: Tawny lion, Cho et al. (2013), C: White lion, Cho et al. (2013), D: Asiatic lion, Mitra et al. (2019). Figure S4. Genome-wide heterozygosity. Panels show heterozygosity genome [file 12915_2019_734_MOESM1_ESM.docx]

**Supplementary Tables**

|  | Assemblies used | Short read data used |
| --- | --- | --- |
| Assemblathon 2 comparisons. BUSCOv3 comparisons, repeats, annotations, and phylogenetic inference | Lion: PanLeo1.0  Tiger: PanTig1.0, DNA Zoo, Armstrong & Kahn et al. 2019  Leopard: PanPar1.0  Iberian Lynx: LYPA1.0  Cheetah: Aci_jub_2  Puma: PumCon1.0, DNA Zoo  Domestic Cat: Felis_catus_9.0 | NA |
| Heterozygosity | Lion: PanLeo1.0  Tiger: PanTig1.0, DNA Zoo, Armstrong & Kahn et al. 2019  Amur leopard: Kim et al. (2016) | Lions: PanLeo1.0 (illumina), SRR836361,SRR836370, SRR5435822  Tiger: Armstrong & Kahn et al. 2019, Cho et al. 2013  Amur leopard: SRR3042211  Malayan tiger: Armstrong & Kahn 2019  Bengal tiger: SRR924676  White tiger: SRR1712667  Amur tiger: SRR836306  Snow leopard:SRR836372 |
| PSMC | Lion: PanLeo1.0 | Lions: PanLeo1.0 (illumina), SRR836361,SRR836370, SRR5435822 |
| Runs of homozygosity | Lion: PanLeo1.0 | Lions: PanLeo1.0 (illumina), SRR836361,SRR836370, SRR5435822 |

**Table S1**: Summary of data sources used for analyses

|  | Original | |  | Closed with single read spanning gap | |  | Gaps closed with split reads | | |
| --- | --- | --- | --- | --- | --- | --- | --- | --- | --- |
| Gap (bp) | Count | Estimated Bases |  | Gaps Filled | Gaps Remaining |  | Filled | Remain | Bases Remaining |
| 10 | 14.431 | 144,310 |  | 13,301 | 1,130 |  | - | 1,130 | 11,300 |
| 100 | 20,136 | 2,016,300 |  | 11.286 | 8,877 |  | - | 8,877 | 887,700 |
| 400 | 4,355 | 1,734,000 |  | 1,816 | 2,519 |  | - | 2,519 | 1,007,600 |
| 410 | 4 | 1,640 |  | - | 4 |  | - | 4 | 1,640 |
| 3,000 | 848 | 2,544,000 |  | - | 848 |  | 135 | 713 | 2,139,000 |
| 5,000 | 2,502 | 12,510,000 |  | - | 2,502 |  | 429 | 2,037 | 10,365,000 |
| 10,000 | 309 | 3,090,000 |  | - | 309 |  | 10 | 299 | 2,990,000 |
| 15,000 | 29 | 435,000 |  | - | 29 |  | - | 29 | 435,000 |
| 20,000 | 6 | 120,000 |  | - | 6 |  | - | 6 | 120,000 |
| 25,000 | 2 | 50,000 |  | - | 2 |  | - | 2 | 50,000 |
| 30,000 | 1 | 30,000 |  | - | 1 |  | - | 1 | 30,000 |
| 35,000 | 3 | 105,000 |  | - | 3 |  | - | 3 | 105,000 |
| 40,000 | 2 | 80,000 |  | - | 2 |  | - | 2 | 80,000 |
|  |  |  |  |  |  |  |  |  |  |
| Total | 42,635 | 22,860,250 |  |  |  |  |  | 15,658 | 18,222,240 |

**Table S2**: Details of genome assembly fill in with Oxford Nanopore data.

| Number of scaffolds | PanLeo1.0 | Tiger | Leopard | Iberian Lynx | Cheetah | Puma | Domestic Cat |
| --- | --- | --- | --- | --- | --- | --- | --- |
| Assembly size | 2.4 | 2.4 | 2.6 | 2.4 | 2.4 | 2.4 | 2.5 |
| Number of Scaffolds | 8,061 | 10,077 | 50,377 | 41,700 | 14,383 | 2,175 | 4,508 |
| Scaffold N50 | 136 Mb | 21 Mb | 21 Mb | 1.5 Mb | 67 Mb | 100 Mb | 150 Mb |
| Scaffold L90 | 18 | 109 | 135 | 2,019 | 780 | 26 | 16 |
| Number of contigs | 22,589 | 36,444 | 162,036 | 86,807 | 140,109 | 157,566 | 4,815 |
| Contig N50 | 312 kb | 186 kb | 41 kb | 107 kb | 47 kb | 32 kb | 514.3 kb |

**Table S3**: Comparative assembly statistics from Assemblathon 2 scripts [45] from published *Panthera* and *Felid* genomes.

|  | **Lion (10x)** | **Lion (10x,HiC)** | **PanLeo1.0 (10x, HiC, Nanopore)** |
| --- | --- | --- | --- |
| Complete | 3,942 | 3,944 | 3,944 |
| Single-copy | 3,918 | 3,920 | 3,920 |
| Duplicated | 24 | 24 | 24 |
| Fragmented | 84 | 78 | 78 |
| Missing | 78 | 82 | 82 |
| % Complete and single-copy | 96.1% | 96.1% | 96.1% |

**Table S4**: BUSCOv3 scores for assembly completeness of the three assembly phases of the African lion genome.

|  | PanLeo1.0 | Tiger | Leopard | Iberian Lynx | Cheetah | Puma | Domestic Cat |
| --- | --- | --- | --- | --- | --- | --- | --- |
| Complete BUSCOs | 3,944 | 3,935 | 3,919 | 3,871 | 3,845 | 3,827 | 3,913 |
| Single-copy | 3,920 | 3,911 | 3,887 | 3,853 | 3,834 | 3,813 | 3,896 |
| Duplicated | 24 | 24 | 32 | 18 | 11 | 14 | 17 |
| Fragmented | 78 | 87 | 92 | 130 | 147 | 143 | 85 |
| Missing | 82 | 82 | 93 | 103 | 112 | 134 | 96 |
| % Complete | 96.1% | 95.9% | 95.5% | 94.3% | 93.7% | 93.2% | 94.8% |

**Table S5**: Comparative BUSCO scores between *published* Panthera *and* *Felid* assemblies.

| **Assembly** | **LINE** | **SINE** | **LTR** | **DNA** | **Unclassified** | **SmRNA** | **Others** | **Total (%)** |
| --- | --- | --- | --- | --- | --- | --- | --- | --- |
| Lion (10x) | 889,658 | 1,468,544 | 315,693 | 342,778 | 13,913 | 1,099,269 | 1,018,633 | 42.30% |
| Lion (10x, H-iC) | 891,625 | 1,469,034 | 315,965 | 342,983 | 13,524 | 1,099,584 | 1,018,250 | 42.33% |
| PanLeo1.0 (10x, Hi-C, Nanopore) | 890,292 | 1,469,373 | 317,748 | 338,708 | 17,853 | 1,099,878 | 1,016,325 | 42.45% |

**Table S6**: Repeat element statistics for the three lion *de novo* genome assemblies generated in this study.

| **Species** | **LINE** | **SINE** | **LTR** | **DNA** | **Unclassified** | **SmRNA** | **Others** | **Total(%)** |
| --- | --- | --- | --- | --- | --- | --- | --- | --- |
| PanLeo1.0 | 890,292 | 1,469,373 | 317,748 | 338,708 | 17,853 | 1,099,878 | 1,016,325 | 42.45 |
| Leopard | 944,218 | 1,564,679 | 330,933 | 354,148 | 14,427 | 1,184,163 | 1,090,064 | 40.97 |
| Tiger | 892,411 | 1,467,533 | 318,598 | 343,750 | 16,369 | 1,097,200 | 1,008,596 | 42.20 |
| Domestic cat | 891,936 | 1,555,928 | 318,675 | 346,001 | 19,497 | 1,183,922 | 1,036,956 | 42.04 |

**Table S7**: Repeat element statistics for various Panthera assemblies and the domestic cat (Felis_catus_9.0).

| **Assembly** | **Total genes annotated** | **Average intron size (bp)** | **Average exon size (bp)** | **Average exons per gene** |
| --- | --- | --- | --- | --- |
| **Lion (10x)** | 20,616 | 3,406 | 1,337 | 7 |
| **Lion (10x, HiC)** | 19,280 | 3,205 | 1,348 | 8 |
| **PanLeo1.0 (10x, HiC, Nanopore)** | 19,258 | 3,193 | 1,383 | 8 |

**Table S8**: Annotation statistics for the three lion *de novo* assemblies generated in this study from the JCVI program.

| **Assembly** | **Total genes annotated** | **Average intron size (bp)** | **Average exon size (bp)** | **Average exons per gene** |
| --- | --- | --- | --- | --- |
| Lion (PanLeo1.0) | 19,258 | 3,193 | 1,383 | 8 |
| Tiger (Cho et al. 2013) | 20,222 | 3,352 | 1,339 | 7 |
| Tiger (Armstrong & Kahn et al. 2019) | 20,261 | 3,045 | 1,401 | 8 |
| Tiger 3 (Cho et al. 2013, DNA Zoo) | 17,953 | 3,447 | 1,281 | 7 |
| Leopard (Kim et al. 2016) | 20,864 | 3,476 | 1,335 | 7 |
| Leopard (Kim et al. 2016, DNA Zoo) | 19,750 | 3,348 | 1,360 | 8 |
| Domestic Cat | 17,111 | 3,197 | 1,344 | 8 |

**Table S9**: Annotation statistics for *Panthera* genome assemblies and the domestic cat (felCat9) using jcvi.

| **Assembly** | **Heterozygosity** | **Number reads mapped** | **Avg. depth** |
| --- | --- | --- | --- |
| Tiger (Cho et al.2013) | 0.0010 | 566,730,100 | 35.6 |
| Tiger (Cho et al. 2013, DNA zoo) | 0.0010 | 566,730,225 | 35.6 |
| Tiger (Armstrong & Kahn et al. 2019) | 0.0010 | 570,020,595 | 35.1 |
| Lion (10x-“Brooke”) | 0.0013 | 869,811,746 | 46.0 |
| Lion (Dovetail, 10x, nanopore-“Brooke”) | 0.0013 | 869,867,776 | 46.0 |

**Table S10**: Observed heterozygosity statistics from various assembly versions of the lion (mapped to the “10x only” and “PanLeo1.0) and tiger (from Cho et al. 2013, and the upgraded DNAZoo tiger assembly).

| **Individual** | **Heterozygosity (mapped to self)** | **Number reads mapped** | **Avg. depth** | **Heterozygosity (mapped to felCat9)** | **Number reads mapped** | **Avg. depth** |
| --- | --- | --- | --- | --- | --- | --- |
| PanLeo1.0 | 0.00120 | 869,867,776 | 46.0 | 0.0009 | 866,802,126 | 45.5 |
| Tawny lion (Cho et al. 2013) | 0.0007 | 801,148,358 | 30.1 | 0.0006 | 797,619,906 | 29.8 |
| White lion (Cho et al. 2013) | 0.0068 | 1,416,024,084 | 53.2 | 0.0056 | 1,409,255,185 | 52.5 |
| Asiatic lion | 0.00019 | 516,572,489 | 23.4 | 0.0008 | 542,324,542 | 22.4 |
| Amur leopard (Kim et al. 2016) | 0.0006 | 1,829,792,054 | 37.0 | 0.0005 | 908,350,242 | 37.3 |
| African leopard (Kim et al. 2016) | 0.0005 | 370,623,557 | 15.0 | 0.0005 | 367,446,294 | 15.3 |
| Malayan tiger (Armstrong et al. 2019) | 0.0010 | 570,020,595 | 35.1 | 0.0008 | 568,170,003 | 34.8 |
| Bengal tiger (Cho et al. 2013) | 0.0009 | 839,375,249 | 31.9 | 0.0009 | 834,272,013 | 31.7 |
| Amur tiger (Cho et al. 2013) | 0.0009 | 727,692,050 | 29.8 | 0.0007 | 721,809,185 | 29.5 |
| White tiger (Cho et al. 2013) | 0.0009 | 727,134,421 | 27.8 | 0.0008 | 723,139,361 | 27.6 |
| Snow leopard (Cho et al. 2013) | N/A | N/A | N/A | 0.0004 | 823,300,505 | 30.2 |

**Table S11**: Heterozygosity (observed) from various *Panthera* individuals when mapped to respective species genome (i.e. lions were mapped to PanLeo1.0, tigers mapped to DNAZoo tiger assembly) genome compared to when mapped to the domestic cat.

| Individual | Bin size | Number of ROH segments | Length of genome | % of genome |
| --- | --- | --- | --- | --- |
| Asiatic | 10kb-100kb | 10,286 | 472,910,162 | 20.6% |
|  | 100kb-1MB | 2,471 | 386,050,440 | 16.8% |
|  | 1MB+ | 0 | NA | NA |
| “Brooke” | 10kb-100kb | 992 | 43,499,165 | 1.9% |
|  | 100kb-1MB | 782 | 215,905,746 | 9.4% |
|  | 1MB+ | 69 | 204099803 | 8.9% |
| White | 10kb-100kb | 5,815 | 245,456,102 | 10.7% |
|  | 100kb-1MB | 1,292 | 220,851,190 | 9.6% |
|  | 1MB+ | 0 | NA | NA |
| Tawny | 10kb-100kb | 2,080 | 74,869,973 | 3.3% |
|  | 100kb-1MB | 1,320 | 470,774,427 | 20.5% |
|  | 1MB+ | 255 | NA | 17.9% |

**Table S12:** Lengths of runs of homozygosity across various lion genomes using PanLeo1.0 as reference.

**Supplementary Figures**

**
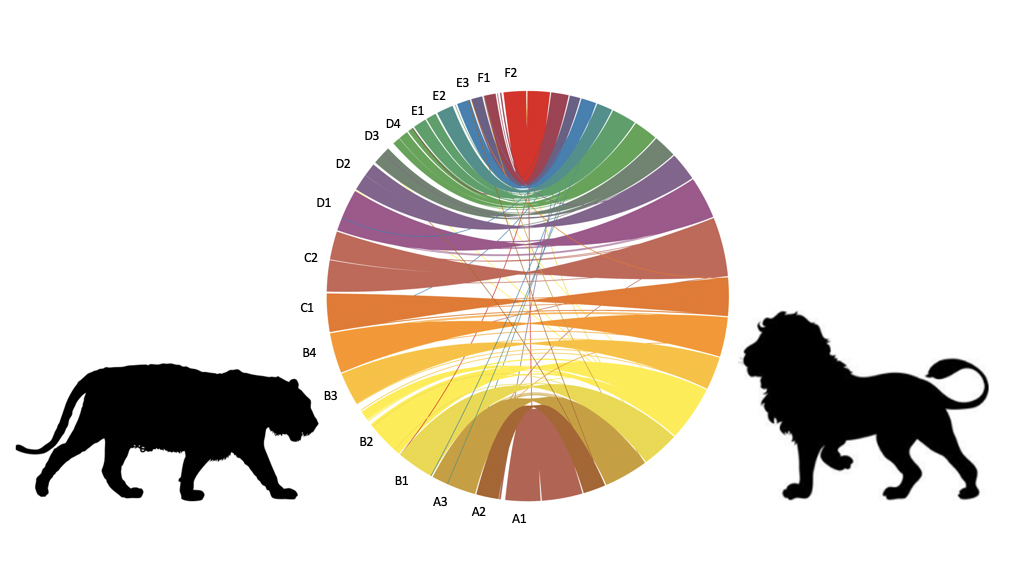
**

**Figure S1:** Circos plot of alignments between tiger (left) and lion (right) chromosomes. Colors represent different chromosomes with bottom chromosome (shown in dark brown) representing A1.

**
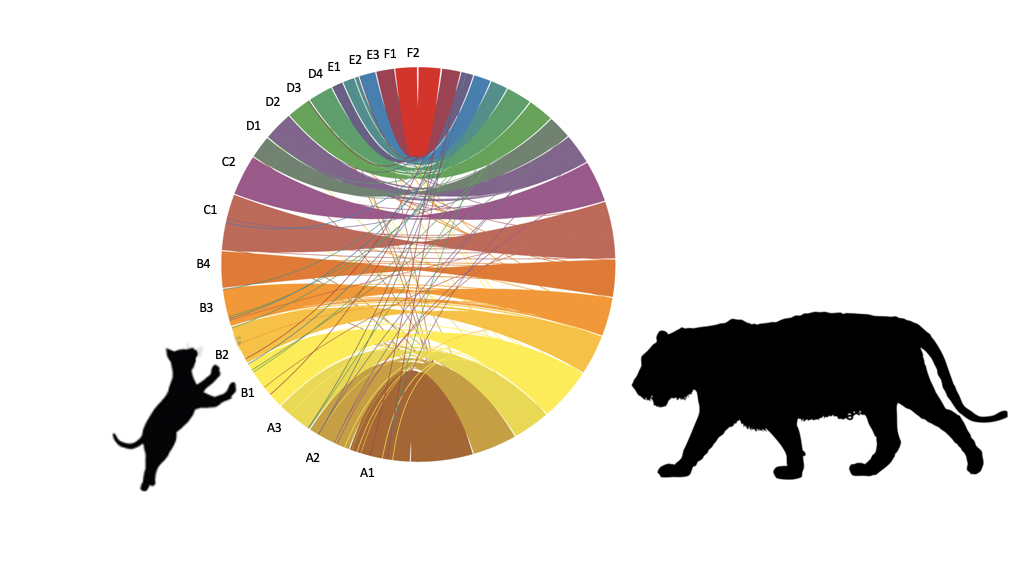
**

**Figure S2:** Circos plot of alignments between tiger (right) and domestic cat (left) chromosomes. Colors represent different chromosomes with bottom chromosome (shown in dark brown) representing A1.


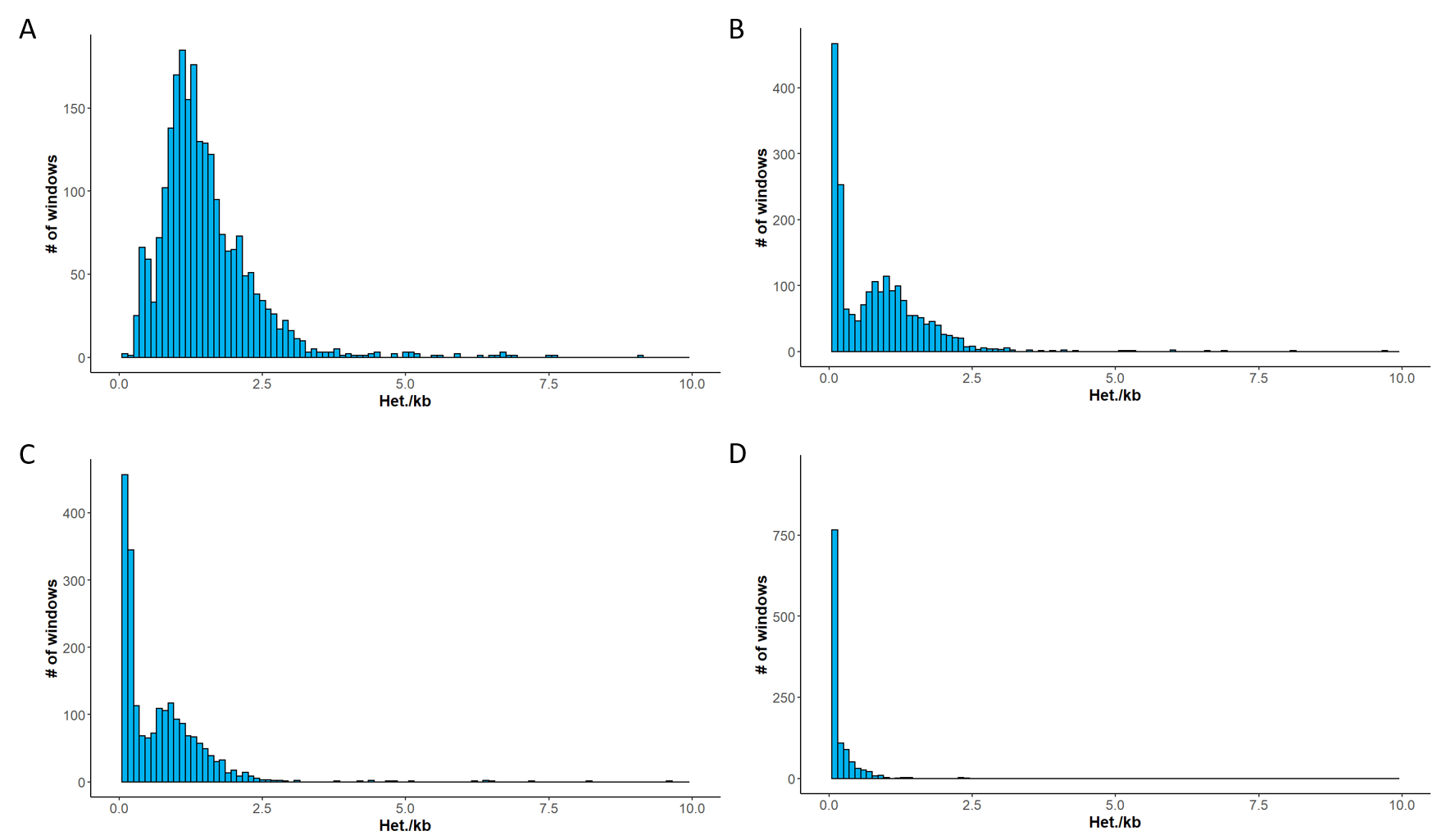


**Figure S3**: Histograms of per window heterozygosity. Graphs skewed more left represent individuals with more windows having lower heterozygosity on average. A: Lion from this study, PanLeo1.0, B: Tawny lion, Cho et al. (2013), C: White lion, Cho et al. (2013), D: Asiatic lion, Mitra et al. (2019).


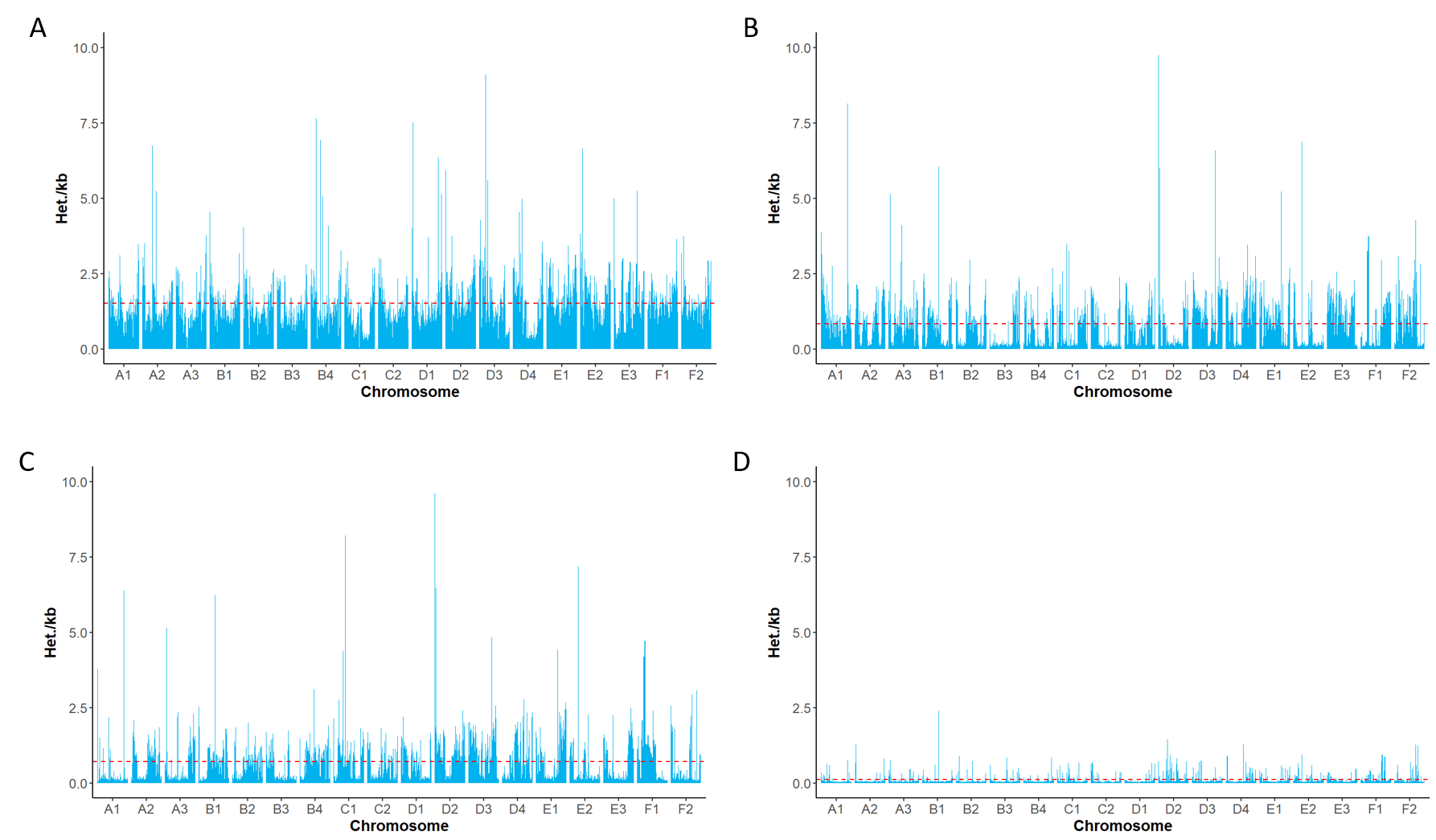


**Figure S4:** Genome-wide heterozygosity. Panels show heterozygosity genome-wide in non-overlapping 1Mb bins. A: Lion from this study, PanLeo1.0, B: Tawny lion, Cho et al. (2013), C: White lion, Cho et al. (2013), D: Asiatic lion, Mitra et al. (2019). Red line represents the mean heterozygosity value genome-wide.


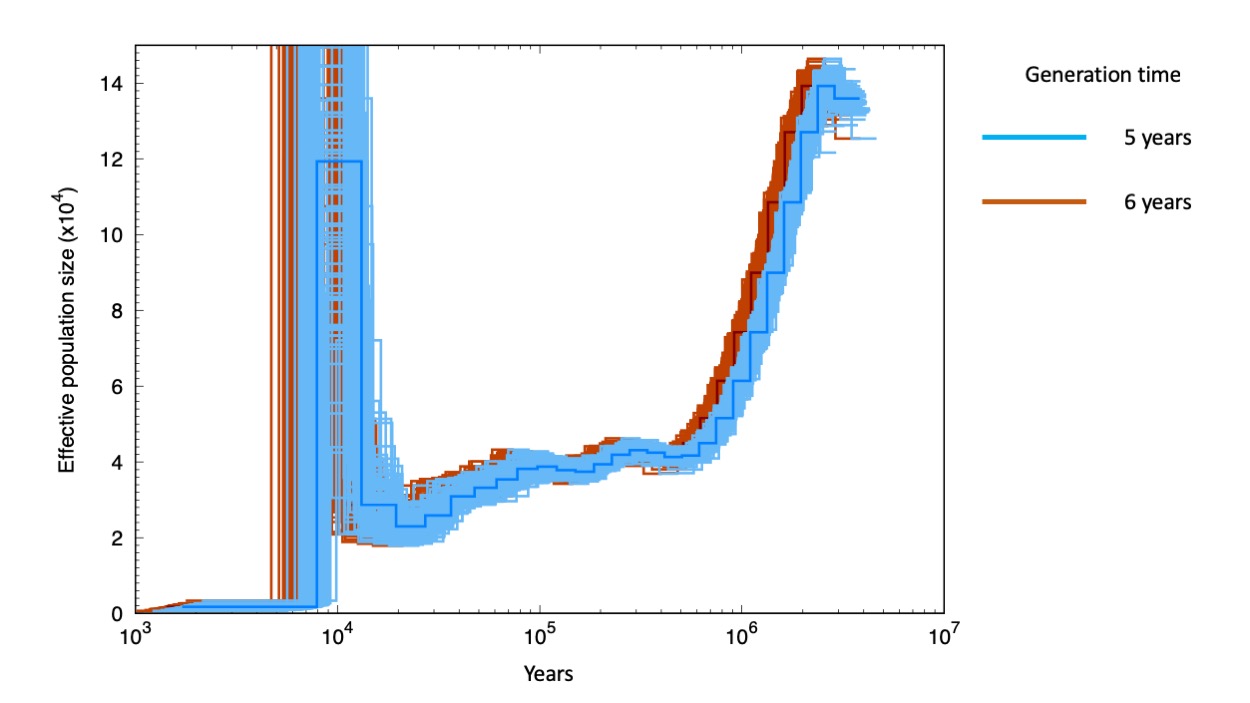


**Figure S5**: Bootstrap PSMC plot comparing generation times of 5 and 6 years using PanLeo1.0 as the reference sequence.
