# Supplementary material for: Long live the king: chromosome-level assembly of the lion (Panthera leo) using linked-read, Hi-C, and long-read data
Source: BMC Biol. 2020 Jan 8;18:3. doi: 10.1186/s12915-019-0734-5 (PMC6950864; doi:10.1186/s12915-019-0734-5)
Supplement: Supplementary file 2 — Additional file 2. Various code used to map and analyze results described in manuscript. [file 12915_2019_734_MOESM2_ESM.docx]

Additional file 2

Code for Submission

Assembly

#run supernova assembler on raw data files from illumine

supernova-1.2.1/supernova run --id lion-170813 --fastqs /path/to/fastq/ --localcores 20 --localmem 250

#generate final assembly files

supernova mkoutput --style=pseudohap –asmdir=assembly-170813/lion-170813/outs/assembly --outprefix=brooke-170813 --headers=short

Quality Assessment-Assemblathon

perl assemblathon_stats.pl assembly.fasta > species_assemblathon.txt

Quality Assessment-BUSCO

python run_BUSCO.py -i assembly.fasta -o species_busco -f -m geno -l mammalia_odb9/ -c 15 -z

Repeat Masking-Prepare for Annotation

#Identify known repeats in genome based on RepeatMasker felid library. Option ‘-nolow’ signals to program to softmask low-complexity repeats so that genes and proteins can be identified during annotation steps.

RepeatMasker/RepeatMasker -pa 10 -gccalc -nolow -species felidae assembly.fasta

#Generate repeat database for identification of unknown repeats

RepeatModeler/BuildDatabase -name species_repeats assemby.fasta.masked

#Run repeat modeler on database generated in previous step

RepeatModeler/RepeatModeler -pa 20 -database awd_repeats

#de novo repeat calling of identified repeats, and merging of de novo and known repeat calls (gccalc calculates GC content across the genome)

RepeatMasker/RepeatMasker -pa 10 -gccalc -nolow -lib RM_4387.TueAug300731172016/consensi.fa.classified species.fasta.masked

Repeat Masking-Repeat Statistics

#repeat above pipeline, but swap ‘-nolow’ with ‘-a’

Annotation

#maker input files freely available from authors upon request

Phylogenetics

#BUSCO parsing scripts available upon request (shared by collaborator)

#align with mafft

parallel mafft --genafpair --maxiterate 10000 {} '>' ./busco_aa_aln/{/.}.aln.fasta

#build trees with raxml

raxmlHPC-PTHREADS-AVX -T 20 -f a -m PROTGAMMAAUTO -p 12345 -x 12345 -# 100 -s ${file} -n ${genename}

#run astral on best tre files

java -jar ASTRAL/astral.5.6.3.jar --input busco_genes_edit2_ml.tree –output busco_species_astral.tree

Mapping for heterozygosity and ROH analysis

#index genome

bwa index -a bwtsw assembly.fasta

#map raw data to reference, convert to bam format, and sort

bwa mem assembly.fasta species.R1.fastq species.R2.fastq | samtools view -bS - | samtools sort - > species.sorted.bam

Generate depth and coverage statistics from bam files

#calculate depth

samtools depth species.sorted.bam |

#calculate how many bases covered by reads in bam file

samtools depth species.sorted.bam | wc -l

#calculate number of basepairs in assembly (for percent coverage calculation)

grep -v ">" assembly.fasta | wc | awk '{print $3-$1}'

Heterozygosity

#create genotype likelihood files for site frequency spectrum estimation from single sample

angsd -i species.sorted.bam -anc reference.fasta -ref reference.fasta -C 50 -minQ 20 -minmapq 30 -dosaf 1 -gl 1 -nThreads 50 -out species_het_angsd

#create actual SFS

realSFS species_het_angsd.saf.id > species.est.ml

#calculate heterozygosity in R

species <- scan(“species.est.ml”)

species[2]/sum(species)

Runs of homozygosity analysis

#binned analysis (supplementary figures S3 and S4)

angsd -GL 1 -doMaf 2 -SNP_pval 1e-6 -doMajorMinor 1 -only_proper_pairs 1 -minQ 15 -bam species.sorted.bam -o species_angsd_homozygosity1

#length of homozygosity runs (Figure 5)

#restrict to desired chromosomes (autosomes)

samtools view -b species.sorted.bam scaffold.name.fasta -o species_scaffoldname.sorted.bam

#call variants using bcftools

bcftools mpileup -Ou -f species.assembly.fasta species.sorted.bam | bcftools call –skip-variants indels -Oz -mv > species.bcfcalls.vcf.gz

#estimate ROH using bcftools

bcftools roh -G30 –AF-dflt 0.4 species.bcfcalls.vcf.gz
